# Hierarchical Event Descriptors (HED): Semi-Structured Tagging for Real-World Events in Large-Scale EEG

**DOI:** 10.3389/fninf.2016.00042

**Published:** 2016-10-17

**Authors:** Nima Bigdely-Shamlo, Jeremy Cockfield, Scott Makeig, Thomas Rognon, Chris La Valle, Makoto Miyakoshi, Kay A. Robbins

**Affiliations:** ^1^Qusp Labs, QuspSan Diego, CA, USA; ^2^Department of Computer Science, University of Texas at San AntonioSan Antonio, TX, USA; ^3^Swartz Center for Computational Neuroscience, University of California, San DiegoSan Diego, CA, USA

**Keywords:** EEG, tags, real-world imaging, event-rich, event ontology

## Abstract

Real-world brain imaging by EEG requires accurate annotation of complex subject-environment interactions in event-rich tasks and paradigms. This paper describes the evolution of the Hierarchical Event Descriptor (HED) system for systematically describing both laboratory and real-world events. HED version 2, first described here, provides the semantic capability of describing a variety of subject and environmental states. HED descriptions can include stimulus presentation events on screen or in virtual worlds, experimental or spontaneous events occurring in the real world environment, and events experienced via one or multiple sensory modalities. Furthermore, HED 2 can distinguish between the mere presence of an object and its actual (or putative) perception by a subject. Although the HED framework has implicit ontological and linked data representations, the user-interface for HED annotation is more intuitive than traditional ontological annotation. We believe that hiding the formal representations allows for a more user-friendly interface, making consistent, detailed tagging of experimental, and real-world events possible for research users. HED is extensible while retaining the advantages of having an enforced common core vocabulary. We have developed a collection of tools to support HED tag assignment and validation; these are available at hedtags.org. A plug-in for EEGLAB (sccn.ucsd.edu/eeglab), CTAGGER, is also available to speed the process of tagging existing studies.

## Introduction

In traditional EEG experiments, participants in controlled environments react to tachistoscopic presentation of stimuli in at most a few categories. Both the stimulus events and the designed reactions of the participants are stereotyped, and researchers usually record the times and types of these planned events using integer event codes (or “triggers”). Researchers then typically assign laboratory-specific event labels to these event types for in-lab discussion and publication. This strategy presents two obvious difficulties for data sharing. First, event labels are not standard across experiments. Secondly, these labels are not sufficient to document relevant details of the stimulus events, the experiment environment, and the assigned participant task(s) within the data. Most EEG analysis occurs within the context of a single experiment or a small set of parallel experimental conditions, allowing for, at best, isolation of a few effects by statistical techniques. Analysis of a single experiment usually assumes that many experimental details equally affect all cells of each statistical contrast and thus experimenters rarely document these details in an accessible form.

Similar experiments conducted by different researchers, even in controlled laboratory environments, typically have different contextual details, and each experimental session unfolds uniquely. A typical session may have many unique real-world events, for example: the researcher pauses the experiment; the participant repositions in a chair or takes a stretch break; the experimenter renews the gel for a detector. The researcher usually identifies and manually excludes such sections of data from analysis based on verbal or written notes that later analysts may not have access to, since they are not typically stored with the data or properly identified for later retrieval. Data around such events are usually considered to contribute only “noise” to the data, but considered together, may indeed contain useful latent information about neurocognition not explored in the original data analysis but potentially amenable to large-scale analysis/meta-analysis.

When science moves from the laboratory to the event-rich environment of the “real world,” annotation of both planned and unplanned events is even more important and complex. We live in a world in which hundreds of stimulus streams vie for our attention. Just as our perceptual system filters out the “irrelevant” while focusing on the “relevant,” the experimenter cannot annotate every possible event detail and must apply an “annotation filter” for relevance. In analyzing a driving perturbation experiment, for example (Chuang et al., 2012, 2014), the experimenters marked as events only the onsets of the perturbations and the onsets of the driver responses. However, their methods for defining these events varied considerably across experiments.

Cross-experiment studies and meta-analysis often requires documentation that is more detailed. Not only planned stimulus presentations and requested participant responses affect driver experience and brain dynamics, but participant anticipation and response to myriad environmental events may likely affect the recorded EEG and other behavioral and psychophysiological data streams. For example vehicle movements toward and away from street signs, pedestrians, speed limit signs, expected and unexpected environment features, as well as changes in driving course and lane topography might be relevant in evaluating neural responses.

Driving experiments are one of the more controlled current real or virtual world experimental paradigms. Experiments in which participants walk through the real world (Debener et al., 2012; Aspinall et al., 2015) may include a variety of planned and unplanned encounters with other people and objects, all of which undoubtedly affect the recorded EEG and other data. These events may thus affect data analysis and may inform unforeseen analyses exploiting the richness of the data—but only if experimental events have been annotated in sufficient detail.

Another difficulty in experiment event annotation is specification of the level of detail at which to annotate the events. Should the researcher always record stimulus size, location, shape, color, texture, and lighting? If this information is available for one experiment and not for another, how can one compare their results? The variety and complexity of events in nature make it impossible to pre-specify a complete event description vocabulary. Yet, without specification of some uniform vocabulary, slight differences in specification may destroy the ability of automated procedures to identify event similarities across experiments or to recognize unforeseen linkages that may exist between event attributes and EEG or other data features.

In 2013, we introduced the Hierarchical Event Descriptor (HED) system (Bigdely-Shamlo et al., 2013a) for recording detailed descriptions of experimental events and storing them with the data. We annotated a number of EEG data collections using HED 1.0 and made them publicly available at headit.org. However, as we began to analyze more “real-world” experimental scenarios and to perform meta-analysis across data collections, we found the HED 1.0 semantics insufficient to express the complicated interactions we encountered. Here, we introduce HED 2.0, a considerable extension of the HED 1.0 system to allow descriptions of the much larger range of events of interest contained in real and virtual world EEG experiments. HED 2.0, combined with our proposed EEG Study Schema (ESS) and containerization tools (Bigdely-Shamlo et al., 2016), create a tool and standards ecosystem that make it possible to store and interrogate data in a large or small collection of similar or diverse EEG studies. The goal of the HED/ESS system is to allow researchers to obtain new information about brain dynamics supporting human experience and behavior not easily obtainable from any one experiment by exploiting information still buried in the accumulating stores of carefully collected EEG and related data. While HED/ESS in combination is specific to EEG and related brain imaging technologies such as fMRI and MEG, HED itself is independent of ESS and is applicable for general annotation in any event rich environment.

This paper describes the evolution of the HED system through Versions 1.0 and 2.0, our development of associated tools, and our experiences in annotating large collections of EEG data. Section The HED 2.0 Structure for Event Annotation gives an overview of the HED 2.0 framework and its differences from HED 1.0. Section Annotation Use Cases and Supporting tools describes our founding use cases and the tools we have developed to support them, reporting experiences in annotating a large multi-collection repository of EEG data. Section HED 2.0 as an Ontology or as Linked Data describes HED in the larger context of potentially more general ontologies and linked data representations. Section Discussion offers some concluding remarks about the future development of HED, its potential for sharing of functional imaging data, and the relationship of HED to other efforts in this area. All of the tools described in this paper are available via Github repositories linked to hedtags.org. Researchers interested in annotating their data using HED and associated tools, should begin by reading *A strategy guide for HED Annotation* guide provided as Supplementary Material with this paper as well as the extensive tool documentation provided at the HED website.

## The HED 2.0 structure for event annotation

The goal of HED 1.0 was to provide a flexible vocabulary for annotating events in EEG laboratory experiments. HED 1.0 was based loosely on CogPO (Turner and Laird, 2011), which is organized along three orthogonal descriptors: stimulus, instruction, and participant response. CogPO has a large number of predefined experimental paradigms allowing users to place an experiment within the contextual taxonomy of large databases such as BrainMap. The purpose of HED, on the other hand, is much more fine-grained. The goal is to annotate individual events for performing large-scale automated analysis and meta-analysis across collections of EEG studies. The requirements in such applications are quite different from the use cases envisioned for CogPO. HED is based on the assumption that a single dataset may contain thousands of events, and that the experimental nuances of each data set and even of each event may vary. These use cases drive the need for a structured, yet flexible vocabulary.

### HED 1.0 structure and limitations

HED 1.0 was a tree-structured vocabulary that allowed users to extend the leaves in certain directions. The top-level annotations for HED 1.0 were */Paradigm, /Time-locked event/Stimulus, /Time-locked event/Response, /State, /Participant, /Context*, and */Custom*. The first three items correspond to the orthogonal elements of the CogPO ontology, while the remaining items document the context of the experiment.

We were able to annotate a number of EEG collections using HED 1.0, These were made public through the UCSD HeadIT project (headit-beta.ucsd.edu). However, when we began to annotate complex experiments conducted outside a controlled laboratory environment, we found that HED 1.0 did not have the semantics needed to describe the experimental and environmental events and event contexts for experimental protocols not based on simple stimulus-response paradigms. Since HED 1.0 maintained a hierarchical strategy, descriptive attributes such as stimulus color proliferated in various portions of the hierarchy. The difficulties we encountered in applying HED 1.0 included:

- Many events may not be directly associated with particular stimulus presentations or participant responses (e.g., the participant in a driving experiment might see pedestrians, buildings, and other vehicles during driving, all peripheral to the driving task).
- Participant states may occur independently of those explicitly manipulated in the experiment (e.g., changes in the participant's attention, fatigue, or stress).
- Participants may fail to perceive some presented items (e.g., the subject may not actually see a speed limit sign displayed in a driving experiment, as evidenced by their subsequent behavior, or by later self-report).
- Items may have multimodal sensory representations (e.g., in a real world driving experiment, the subject might hear as well as see other vehicles).
- Different presentations associated with the same stimulus category may not be associated with equivalent EEG dynamics (e.g., EEG dynamics induced by seeing a 2-D picture of an apple, a 3-D representation of an apple in virtual reality, or an actual apple).
- A typical event associated with a complex scenario may require descriptions of multiple objects (e.g., during driving, the experience of being in a traffic jam).

Some of these difficulties in using HED 1.0 stemmed from the lack of orthogonality in the definitions of item attributes. For example, attributes such as color, size, and location can apply to virtually any item and hence repeat in many of the leaf nodes. We concluded that attributes should instead be orthogonal to the items they describe and therefore should appear as separate nodes under a top-level Attribute node in the hierarchy.

Another difficulty was that events sometimes required descriptions of multiple items with multimodal representations. Thus, Sensory presentation should be a top-level item. Other complexities arise for experiments involving multiple participants and experiments that encompass multiple experimental contexts. In one set of experiments, we helped tag, participants began by walking on a laboratory treadmill viewing a virtual reality scenario. They then performed the same tasks outdoors in a park environment. The lack of embedded unit specifications was also a barrier to mining similar events across data collections. Finally, real-world experiments may require annotation of many events peripheral to the central participant task. To describe such events, the tag vocabulary must be capable of describing all relevant aspects of these events.

### HED 2.0—overall structure

To address the concerns articulated in the previous section, we revised HED 1.0, giving it a more complete and orthogonal hierarchical structure. We refer to this scheme as HED 2.0 and maintain the current HED 2.0 specification in Wiki format at hedtags.org/schema. Methods are available to export the HED 2.0 schema from wiki to XML and JSON formats for use in HED tools.

**Figure 1:**
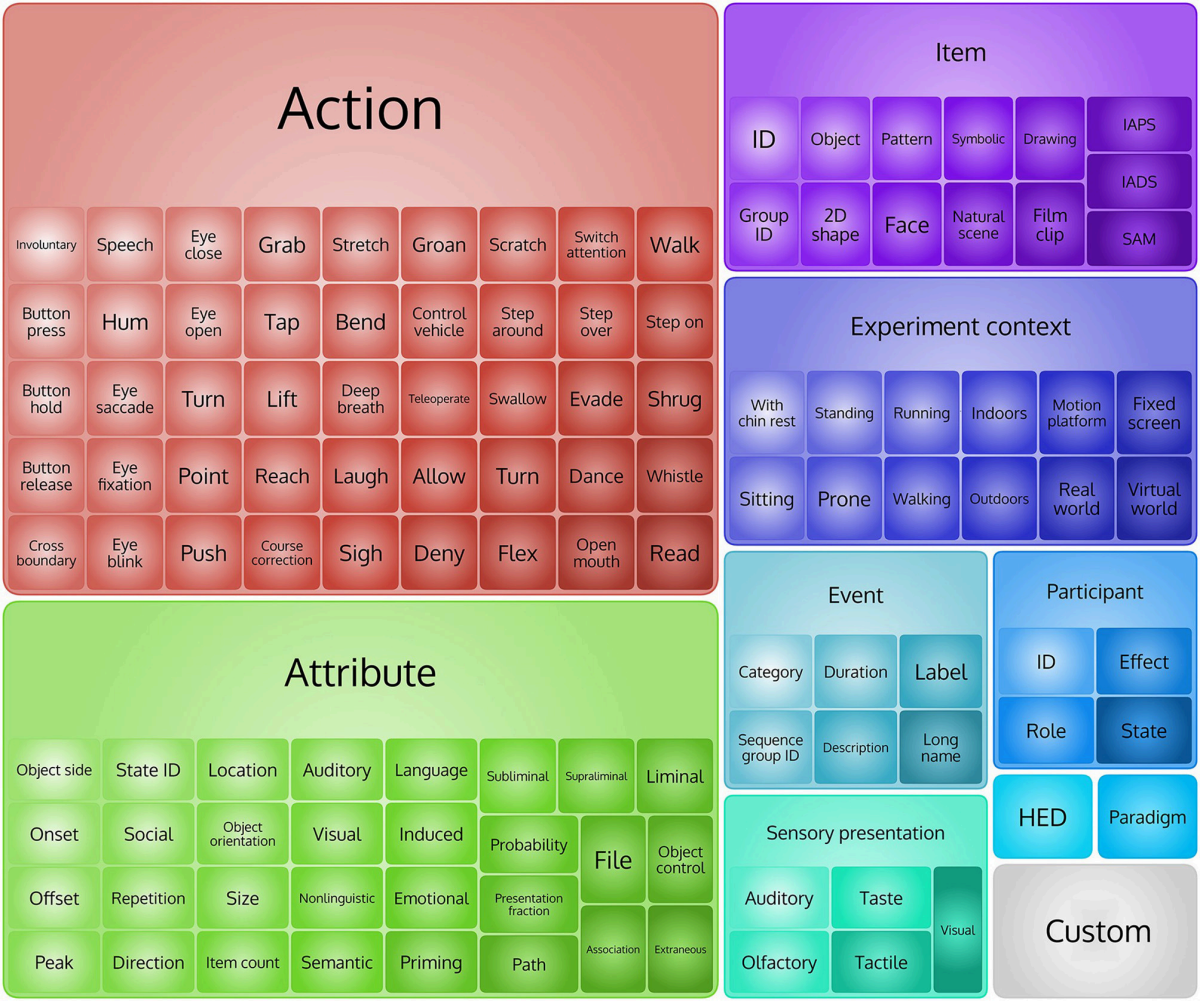
**Top levels tags in HED 2 hierarchy**.

The HED 2.0 schema has the following top-level elements (Figure 1):


      /Event                     /Participant
  
      /Item                      /Experiment
                                  context
  
      /Sensory presentation      /Paradigm
  
      /Attribute                 /HED
  
      /Action                    /Custom


HED tags may appear in the overall description of an experiment or as annotations associated with individual events. Experiment-related tags mainly come from the /Experiment context and the /Paradigm sub-trees. Descriptive elements appear under the /Attribute sub-tree and can modify any aspect of the hierarchy. The /Attribute sub-tree contains many more attributes than were present (across multiple branches) in the defined HED 1.0 hierarchy. HED 2.0 also contains unit classes including time, physicalLength, angle, and frequency, each with associated allowed and default values. The unit classes as well as attributes such as the data types are part of the HED 2.0 specification, allowing HED tools to perform automated consistency checking of tagged events. Events can also have onset, offset, and duration attributes. These allow HED 2.0 to describe events that are instantaneous or that persist for some duration. There are 949 predefined tags in HED 2.0: 10 top-level tags, 314 tags at level two (2), 213 at level three (3), and 417 tags in levels four (4) to seven (7).

In HED 2.0, every event is required to have /Event/Category and /Event/Label tags (plus their children). A child of the Label tag is a short (less than 20 character) identifier (e.g., “Button press”) meant to help researchers refer to events of the same type within a study in a convenient manner. Often the label corresponds to the label that researchers used to identify events of this type within their laboratories. Automated processing usually ignores /Event/Label, since by definition its content scope is limited to the current study only, meaning that events labeled similarly across multiple studies may be different in nature. The child of /Event/Description node, which is a human-readable description of what this event represents, is also meant to be ignored in automated processing.

Children of the top-level element /Event/Category include Initial context, Participant response, Technical error, Participant failure, Environmental, Experimental stimulus, Experimental procedure, Incidental, Miscellaneous, and Experimental control. The event subcategory /Event/Category/Environmental can document a variety of environmental conditions, relevant or not relevant to the experiment (e.g., in an outdoor experiment, hearing noisy airplanes flying overhead). In experiments taking place in outdoor environments, researchers may use this subcategory to encode unplanned interactions with other people using /Event/Category/Incidental or /Event/Category/Miscellaneous. The key for successful annotation is to provide a description sufficient to resolve ambiguities. Machine learning algorithms may either attend to or ignore these details; researchers may use the detailed descriptions to explain variability in the data or to exclude portions of the signal from analysis.

### The role of participants in HED 2.0

In addition to making attributes orthogonal to the rest of the hierarchy, HED 2.0 separates specification of participant attributes from the specification of presented stimuli and their perception (or otherwise) by the participant. This organization was motivated by experiments that may include multiple participants and/or (e.g., in real-world experiments) other people who may perform actions relevant to the subject. In addition, laboratory experiments typically present stimuli in a way that strongly constrains actual participant perception of the stimuli (e.g., participant focuses on a visual display in a darkened room). Real-world experiments often have much weaker guarantees about whether a participant actually perceived a given stimulus. The top-level HED 2.0 items under /Participant are ID (default: 1), Role, Effect (to document how the stimulus does or should affect the participant), and State (to document participant level of consciousness, attention, fatigue, etc.). As determining the true effect of a stimulus event on a participant is difficult, researchers may typically use these tags to document an event's intended effect. For example, a researcher might document an event designed to feel rewarding as /Participant/Effect/Cognitive/Reward or (adding a pertinent detail) as /Participant/Effect/Cognitive/Reward/$10.

HED 2.0 also generalizes the specification of actions, decoupling them from the specifications of participants and stimulus presentations. This decoupling allows researchers to document task-irrelevant or involuntary participant actions such as yawning, hiccupping, scratching, etc., that may affect data recording or to describe actions of other agents in the paradigm (such as actions of autonomous cars).

### HED 2.0 example combinations and semantics

HED 2.0 allows detailed documentation of multiple items within an event. The following example describes a situation in which the stimulus consists of two items presented simultaneously to the participant. However, the participant only visually attends to one of the stimulus items, as seen later in the eye tracking data:


    /Event/Category/Experimental stimulus,
  
    /Event/Label/RedFixationCircle,
  
    /Event/Description/Displayed to user a
     red circle for fixation in center of the
     screen and a blue square on the left.
     User sees the red circle,
  
    (/Item/2D shape/Ellipse/Circle,
    /Attribute/Visual/Color/Red,
    /Attribute/Fixation point,
    /Attribute/Location/Screen/Center,
    /Sensory presentation/Visual/Rendering
     type/Screen/2D,
    **/Participant/Effect/Visual),**
    
    (/Item/2D shape/Rectangle/Square,
    /Attribute/Visual/Color/Blue,
    /Attribute/Location/Screen/Left,
    /Sensory presentation/Visual/Rendering
     type/Screen/2D)


Parentheses group tags and enable specification of multiple items and their attributes in a single HED string. Here, both the red circle and the blue rectangle have /Sensory presentation/Visual tags, indicating the items appeared on a screen the participant was viewing. Only the red circle tag contains /Participant/Effect/Visual, indicating the subject saw the circle (e.g., based on eye-tracker information). Currently most of our tagging efforts assume subjects actually see all visible items. As eye tracking becomes more common in EEG experiments, we expect annotators to distinguish more carefully between visibility and perception (either peri- or extrafoveal).

HED 2.0 also allows description of more complicated events using a sentence-like tag grouping syntax. Describing the sentence, “Man ate Fish” requires specifying the order of “Man” and “Fish” in the sentence (to differentiate “Man ate fish” from “Fish Ate Man”). HED 2.0 expresses these semantics using an RDF-like (Subject, Predicate, Object) construct with tilde (~) as separator:


    (/Item/Object/Person ~ /Action/Type/Eat ~
     /Item/Object/Animal/Fish)


The third clause (the object) is not required. For example, the following conveys “Car [is] perturbed”:


    (/Item/Object/Vehicle/Car ~
     /Attribute/Vehicle Control/Perturb)


Another common situation requiring thoughtful annotation is the notion of a “target” stimulus presentation (i.e., a type of stimulus presentation event the participant is set to respond to) vs. a “non-target” stimulus presentation (an event not prompting a participant response) vs. presentation of an “oddball” stimulus (presentation of a stimulus not within any expected stimulus stream and appearing relatively rarely). For example, in a visual oddball experiment, a subject is instructed to press a button only when a chair appears.


    /Event/Category/Experimental stimulus,
    /Event/Description/A picture chair is
     displayed on the screen,
    /Sensory presentation/Visual,
    /Participant/Effect/Visual,
    /Participant/Effect/Cognitive/Target,
    /Item/Object/Furniture/Chair


The “target event,” seeing the chair, may or may not be rare, and thus may or may not elicit a typical “oddball” response. The event annotation may contain the following tags to designate an oddball stimulus presentation:


    /Participant/Effect/Cognitive/Oddball,
    /Attribute/Presentation fraction/0.1


Targets may also elicit a threat level or other cognitive effect, which researchers can annotate based on the meaning of the experiment. In a classical N-back problem meant to test working memory, the “target” is a letter that matches a letter a fixed number of items back in a sequence of letters in a presentation stream (cognitiveatlas.org/task/n-back_task).

Annotators of laboratory experiments generally assume that visual items are presented on a computer screen, which can be designated as:


    /Sensory presentation/Visual/Rendering
     type/Screen


Once annotated collections are available, researchers can extract different data subsets for within- and cross-session analysis as described in the next section.

## Annotation use cases and supporting tools

The simplest use case for annotated data is simply to gather data from multiple studies that share a common characteristic (such as including visual “oddball” stimulus presentations). Providing a summary of the unique tags for each dataset and for each study makes such an operation easy. Once researchers have identified a group of datasets of interest, analysis may proceed in several different directions.

The most important mode of EEG analysis is to extract sets of EEG epochs time-locked to selected classes of events to observe and model differences in EEG patterns induced by event types of interest. Epoch-based analyses may involve computing trial-averaged event-related potentials (ERPs), event-related spectral perturbations (ERSPs) (Makeig, 1993), or other measures averaging across trials in the time or time-frequency domains. Researchers may easily group HED-tagged event-locked epochs or compare epochs based on similarities in several attribute dimensions coded in the event HED tags. Tools such as LIMO (Pernet et al., 2011) facilitate the introduction into a General Linear Model of potentially relevant event dimensions as independent factors (for example the color, size, or intensity of presented target or non-target visual stimuli). Functional connectivity analysis, trial classification, and BCI (brain-computer interface) design also involve extracting and modeling epochs that are time-locked to particular classes of events.

Another application for event tagging is enrichment analysis. Given a large corpus of data with tagged events, one can ask whether particular tags are more likely to be associated with a given data pattern than a random relationship would suggest. Researchers can use statistical approaches such as independent component analysis (ICA) to discover unanticipated relationships between data features and event tag combinations. Such methods allow fruitful experiment designs to expand beyond repeated presentation of a few stimulus stereotypes, instead presenting to participants a wealth of individual stimuli that may vary randomly across some parameter space of interest. A rich space of individually varying events is also characteristic of real-world and complex virtual-world experiments. HED tagging can allow information-based methods to define factors associated with cognitive and brain processes that are not perfectly modeled by any single event tag.

### Types of HED annotations

Three event-tagging processes (*preset, real-time*, and *post hoc* annotation) use different strategies for tagging and require different supporting tools. In traditional EEG experiments (*preset*), researchers define stimulus presentation and participant response events in advance when designing the experimental paradigm and typically encode these events in the EEG data files using experimenter-defined numerical codes. The number of such event codes can range from a handful to a few hundred, and events with the same event code usually appear multiple times during the experiment. In the future, experiment control programs running such experiments could easily place in the data file full HED tag descriptions of each such preplanned event, eliminating the need to translate cryptic data event codes into fully descriptive HED tags after the experiment finishes.

More flexible experiment control programs (e.g., virtual-world simulators) typically generate rich event streams that can also be labeled in real time using an appropriate combination of predefined tags to describe each event (*real-time*). In our experience, a virtual-world experiment application may produce and place in the recorded data hundreds of thousands of uniquely annotated events. The subset of relevant details may differ in each analysis, depending on its goals. Ideally, this automated HED tagging should attempt to document all possible event details for later filtering to support specific analyses. The bandwidth of the generated HED tag data stream will usually occupy only a fraction of the bandwidth of the recorded data.

In a third type of event annotation, researchers may designate and annotate events *post hoc* from preliminary inspection or analysis of the data. In videotaped experiments, for example, researchers may search visually for and then hand-annotate complex video events (e.g., particular participant gestures) that cannot be recognized objectively and tagged during the experiment by the experiment control application. In experiments using eye-trackers, *post hoc* processing of gaze paths and fixation locations may be needed to determine and annotate whether or not a subject viewed presented objects (whose positions have ideally been detailed in the application-generated HED tag stream).

### CTAGGER annotation tool

We have developed several toolsets to facilitate the processes of event annotation, validation, extraction, and search using HED 2.0. In the discussion below, we assume that the EEG researcher has created a small list of event codes from an experiment in an Excel spreadsheet, now a common strategy in EEG research. The HED annotation process in this case would usually consist of mapping these event codes to appropriate HED tags. The actual events that occur in the experiment are often stored in a spreadsheet (say a tab-separated text file), or for users of EEGLAB in the EEG.event structure.

A researcher starting the annotation process with a spreadsheet of event codes would benefit from using CTAGGER (for “community tagger”), an open source Java HED-based annotation tool, created by us, that runs as a standalone program or as a function called from MATLAB (either standalone or as a plug-in tool for EEGLAB). We first released CTAGGER for HED 1.0 several years ago (Rognon et al., 2013) and have substantially modified the tool to support HED 2.0.

**Figure 2:**
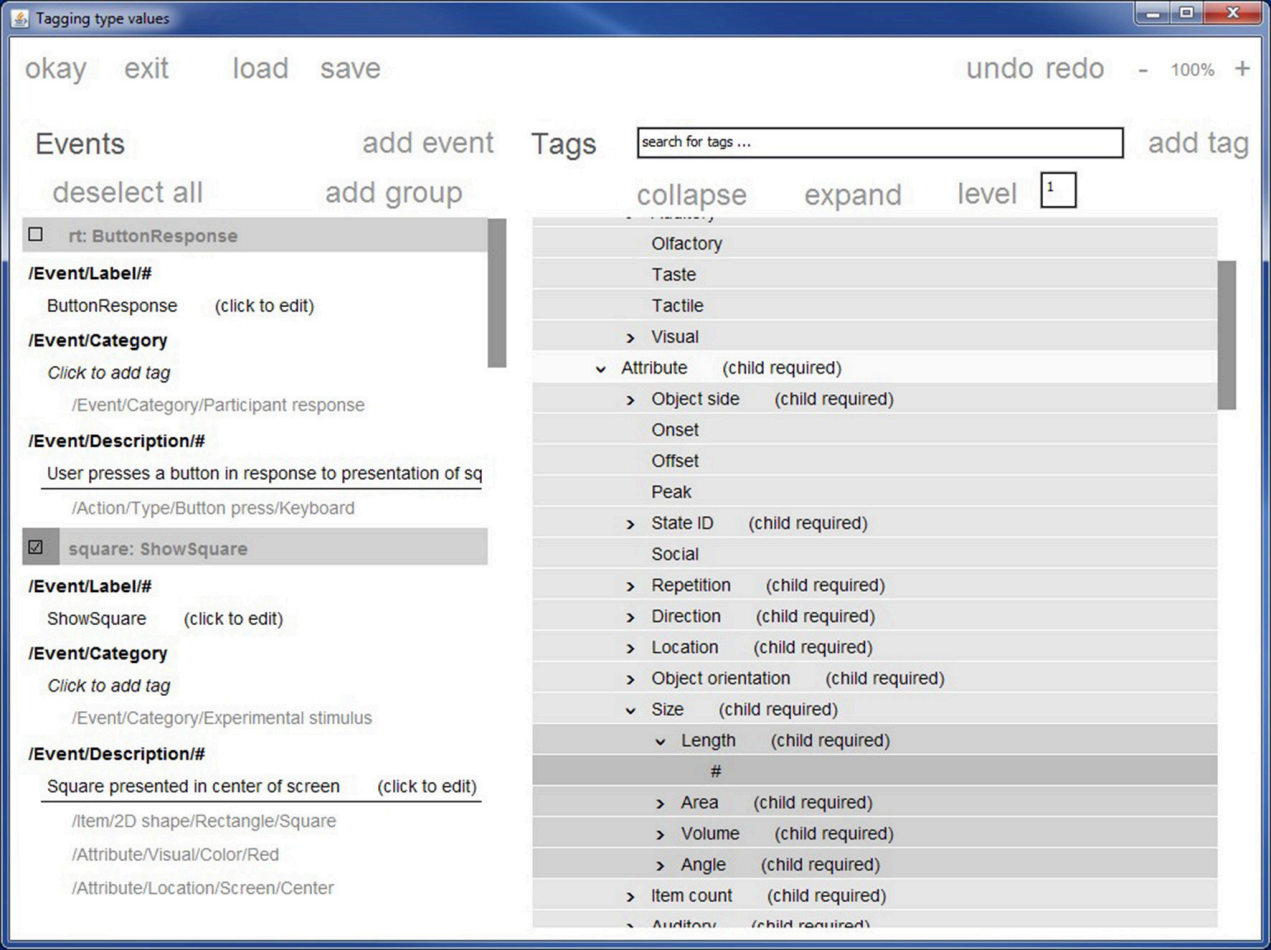
**The CTAGGER graphic user interface for tagging events**. Stimulus presentation events described in the original data only as *rt* and *square* are now tagged with more detail that could prove informative to initial analysis or later meta-analysis.

Figure 2 shows a screen shot of the primary CTAGGER annotation tool window. This window has two main panels: the left panel displays the events or event codes to be tagged, while the right panel displays the hierarchical vocabulary (HED or other conforming vocabulary) from which tags may be selected. Each event or event code has its own section whose header has a checkbox. Users can simultaneously tag multiple event codes by checking their respective boxes in the left panel. CTAGGER adds (associates) the selected tag with each selected event or event code. CTAGGER also supports undo operations as well as removal of a tag from a group of selected codes that all contain the selected tag.

Tags for attributes such as object sizes and presentation durations require a child tag specifying a value. The HED 2.0 schema represents specific values using the # wildcard, usually associating this wildcard with an appropriate unit class (such as length, area, or angle). CTAGGER automatically verifies valid and default units as well as positions where a user must supply a value.

The collapse/expand/level option addresses the difficulties that arise because the HED hierarchy is large and has a somewhat irregular depth structure. Certain levels of the hierarchy are deep (up to seven levels), while other portions of the hierarchy are relatively flat. Users can easily lose context after navigating to a level deep in the hierarchy. Users can thus set a level in the level box, causing CTAGGER to collapse all branches of the hierarchy to the specified level.

The context-search feature helps users quickly navigate to the desired part of the hierarchy based on any part of a word or phrase. As the user types in the box at the top of the right panel, a pull-down menu displays possible matches within the hierarchy and continues to prune the list as the user types more of the desired search term. When the user clicks on a value in the search pull-down or hits enter in the search box, CTAGGER immediately centers the right panel hierarchy on the selected term.

The CTAGGER annotation tool can read a tab-separated file in which each line has a column containing an event identifier and additional columns containing lists of tags. The event identifier can be either an event code or an event latency in the data.

### Validation tools

The HED 2.0 hierarchy is represented in XML and follows a simple schema in which each node consists of a name, a description, and any child nodes. The allowed attributes of a node are required, child required, unique, recommended, takes value, is numeric, position, type, unit class, and predicateType. HED 2.0 specifically requires /Event/Category and /Event/Label and recommends /Event/Description. Examples of unit classes include time, length, area, volume, currency, velocity and jerk. The *HEDTools* collection is available for MATLAB. Some validation tools are also available in Python. Validation options include:

- Verify a HED hierarchy specified in XML against an XML schema
- Convert a MediaWiki formatted text representation of HED into XML
- Verify a list of tags against a valid HED specification in XML
- Map HED 1.0 tags into HED 2.0 tags

The MATLAB *HEDtools* are available via a GUI or as functions that can be called as MATLAB commands. For example, the following call to the function validateTSVTags validates the tags in columns 2 and 3 of a specified tab-separated text file.


  [errors, warnings, extensions, remap] = ...
      validateTSVTags('CIT_events.tsv', ...
                 [2, 3], 'hasHeader', false);


The returned items are cell arrays containing lists of errors, warnings, and tags that are not in the HED specification because they are extensions provided by the user in allowed places. The remap array is a list of unique invalid tags. A researcher can use the remap array to create a correspondence between incorrect tags and corrected tags. Calling remapTSVTags with the remap array then allows users to remap all occurrences to corrected tags in a single step.

### Integration with MATLAB and EEGLAB

Many neuroscientists analyze EEG data using MATLAB. These users can either call the CTAGGER annotation tool from MATLAB directly or can install it as an EEGLAB plug-in. Generally, the MATLAB interface assumes that the EEG data and events are stored in an EEGLAB EEG structure. CTAGGER uses the EEG.event.usertags field to store the tags associated with a particular event code and the EEG.event.hedtags field to store tags specific to an event occurring at a designated latency (for example, HED tags streamed from an experiment control application). CTAGGER also stores the association of tag mappings to event codes in the EEG.etc.tags field. The CTAGGER tools use these mappings internally to allow users to edit previously tagged EEG. The *HEDTools* toolbox allows tagging of a directory/folder containing several datasets or an entire EEGLAB study as well as individual files. Users can create a mapping between event codes and tags and reuse that mapping for an entire study. CTAGGER also has an EEGLAB plug-in function that allows EEGLAB users to tag events using a GUI through the EEGLAB interface. Extraction of data epochs based on HED tags follows the EEGLAB format and can be done either from a tool GUI or from the command line.

### Tagging workflow and the learning curve

The amount of effort required to HED tag a data collection depends on the number of unique event types in the collection. A traditional EEG experiment might have thousands of individual events, but only 10 or so unique event types. An experienced tagger might tag a complete collection of this type in an hour or so. In guiding several novice taggers through the process, we have found that most of the learning curve is in understanding how the vocabulary is organized and what tagging elements should be present to express each event properly. After tagging a few event types, the process usually goes quickly unless something non-standard must be tagged.

The two most common tagging workflows use the toolset in different ways:

#### CTAGGER workflow

1. Read the codes representing unique event types into *CTAGGER* or create the codes using the *CTAGGER* GUI.
2. Tag each code using the GUI. (*CTAGGER* validates selections continuously.)
3. Save the tagged codes in a map file using *CTAGGER*.
4. Use the saved map file to tag the entire collection with one call to the tagdir function.

#### Spreadsheet workflow

1. Start with your unique event codes in a single column of a tab-separated spreadsheet.
2. Manually enter the tags for each code in one or more other columns (usually using a lot of cut and paste operations). Use https://github.com/BigEEGConsortium/HED/wiki/HED-Schema as a guide for the allowable vocabulary.
3. Call validateTSVTags with the spreadsheet file as input. Using the output of this function to find and correct errors. Continue correcting and revalidating to eliminate all errors.
4. Save the validated spreadsheet and then use the spreadsheet with tagdir to tag an entire collection of EEG files.
5. If your actual events, identified by codes and latencies, are in a tab-separated spreadsheet you can call a function to produce a tagged spreadsheet of events using the spreadsheet from Step 4 as a second input.

Thorough event annotation definitely requires (and deserves) iterative processing and thoughtful consideration of what events actually represent. The *HEDTools* tagging infrastructure supports multiple passes through the tagging procedure and allows analysis tools to filter at different levels of detail. In addition to tagging, *HEDTools* also supports creating epochs from tag combinations using tools compatible with the EEGLAB epoching tools.

### Experience with annotation of a large collection

**Figure 3:**
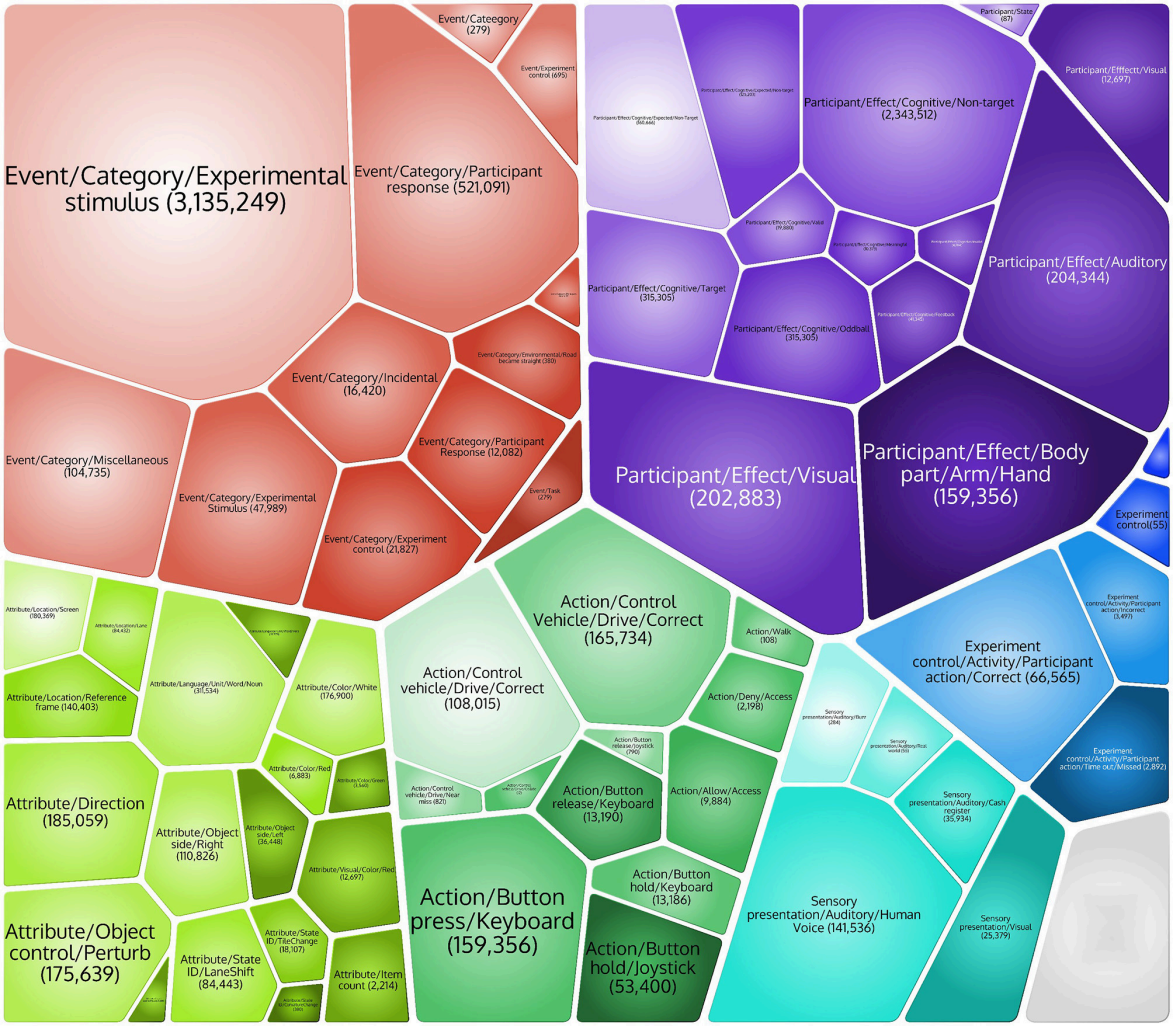
**Major event HED tags and the numbers of event instances matching each tag across 3,836,429 individual events from 18 studies in a data repository project whose construction required and prompted the development of HED 2.0**.

So far, we have tagged millions of events across 1860 datasets from 22 EEG studies. We are also in the process of converting the publicly available HED 1.0 annotated datasets from headit.org to HED 2.0 and ESS 2.0 (Bigdely-Shamlo et al., 2016). Figure 3 shows a visual representation of the relative numbers of occurrences of events in several major categories.

The annotation process has prompted our more careful thinking about the meaning of different events and their potential cognitive effects: Should spotting a particular target in a given experiment design elicit a “threat” or “oddball” response (or both)? How should we identify different types of “cues” (events that cue the occurrence of other events in near future)? What should the event tag descriptions be for stimuli not presented suddenly as a whole (as in most previous EEG laboratory experiments)?

We also asked for comments from researchers outside our laboratories who were using HED tags to annotate their events. We have included some of their comments in the Supplementary Material. The overall sense was that the HED tagging process was fairly straightforward, once they read some examples, and that the tagging process helped them understand their events in a larger context. They suggested the need for a user forum to facilitate community discussion to control how new tags are added and used.

## HED 2.0 as an ontology or as linked data

So far, we have described HED 2.0 as a framework rather than as an ontology. Even if we could create a formal ontology for annotating EEG events, the resulting complex network of relationships between all possible items (in either the real world or some virtual world), would make such an ontology very difficult for users to comprehend and work with. HED 2.0 is, in some sense, an attempt to provide a more palatable interface for large-scale community tagging. Rather than trying to create and visualize a complex ontology, we have taken the approach of hiding the detailed ontology structure from the annotator. The ontology underlying HED 2.0 is quite simple. HED 2.0 has seven top-level classes: item, participant, action, attribute, sensory presentation, paradigm, and *event*. Table 1 shows the top-level relationships. We assume that subclasses of a class have the same relationships as the parent class without enforcing that such a relationship “makes sense.” For example, a visual stimulus object described as /Item/2D shape/Star is unlikely to perform an action best described as /Action/Type/Walk, though our approach allows for such a tag combination. We assume that the pair will not appear together in practice unless somehow the relationship does make sense.

**Table 1:** **Top-level HED 2.0 classes and implied relationships**.

**Classes**	**Relation (→)**	**Classes**
Item, Participant, Event	*isDescribedBy*	Attribute
Attribute	*describes*	Item, Participant, Event
Action	*isPerformedBy*	Item, Participant
Item, Participant	*performs*	Action
Event	*isDescribedBy*	Paradigm
Paradigm	*describes*	Event
Sensory presentation	*presents*	Item
Item	*isPresentedBy*	Sensory presentation

A second aspect of our approach, with respect to mapping to formal ontologies, is that our hierarchy mixes subclasses and properties. For example, in the following piece of the HED 2.0 hierarchy, ID and Group ID are properties, while Object and 2D shape are subclasses:


    /Item
        /ID
        /Group ID
        /Object
           /2D shape ... 


We argue that during the annotation process users do not care about the formal distinction between is-a and has-a relationships. Keeping the property and subclass information together here makes the user's annotation experience less complicated. The HED 2.0 XML specification internally distinguishes these two types of relationship using a *predicateType* attribute (either rdfs:subClassOf for subclasses or a HED-defined isPropertyOf relationship (a subproperty of the rdfs:domain property). By design, this distinction is not visible to the user. The few properties encoded into the hierarchy are identifying in nature (e.g., *ID*). A strong argument for using a semi-structured tagging approach is that most of the anticipated analysis applications can then use prefix matching to extract relevant events, an operation that is easy to implement and simple to understand.

Another fundamental difference between using HED and working directly with an ontology is that users can extend HED at any leaf node and at any node with an *Extension allowed* attribute without changing the relationships or inheritance properties of nodes higher in the hierarchy. Thus, users can fill in greater levels of detail and extend to new areas. Extension tags that appear commonly can then be added to the hierarchy as permanent nodes if they prove to be of more general use and/or value.

In spite of differences between HED and formal ontologies, RDF statements can be produced for event types or instances using the abovementioned relationships. For example, for event instance E identified with the URI hedtags.org/schema/v2/EventInstance/123 and associated with HED string HS identified with URI H = hedtags.org/schema/v2/HEDString/HS' where HS' is a normalized version of HS (with tags arranged in a standard order to create a unique string representation) we have (in subject predicate object format):


              E rdfs:type H
    [If E is an event type, the relationship
     will be instead rdfs:subClassOf]


…followed by a number of statements that describe H. These statements could be dynamically produced based on HED tags and their co-occurrences in tag groups (see Table 1): if H has tags identified by URIs T1 and T2, these statements will include the relationship between H and these tags, along with relationships that may exist between T1 and T2. In case of /Event/Label and /Event/Description, these relationships are rdfs:label and rdfs:comment:


      H rdfs:label [label string extracted
      from Event/Label tag]
      H rdfs:comment [description string
      extracted from Event/Description tag]


Converting between HED and formal ontology and RDF representations should allow formal ontology tools to operate on HED annotated data and for HED annotated data to be included in larger databases using these formal structures.

## Discussion

HED is unique in its focus on events. Many of the current efforts in sharing neuroimaging data (fMRI, EEG, and MEG), such as COINS Neuroimaging Suite (Scott et al., 2011) and Open fMRI (Poldrack et al., 2013; openfmri.org) focus on experiment-level annotation. COINS has powerful tools for tracking patients through clinical studies and supports sophisticated querying. COINS does not explicitly deal with experiment event annotation. Open fMRI documents events, which in this context are mainly participant responses, as behavioral data stored in tab-separated text files. Open fMRI specifies that events should be documented, but does not provide or require the use of a standardized vocabulary. Open fMRI also provides a standardized processing pipeline and group statistical analyses. Recently, the Open fMRI data structures have been formalized as the Brain Imaging Data Structure (BIDS) specification (Gorgolewski et al., 2015). This specification describes a directory structure and the naming scheme for files within the directories. Events are specified in tab-separated files with the first columns (onset and duration in seconds) being mandatory. Researchers may use other columns to specify other event properties, but BIDS imposes no other requirements for specifying or detailing the nature of experimental events. Thus, HED annotation is compatible with BIDS and could be easily used to standardize the annotation of events in the fMRI community. Tags to annotate experimental conditions specific to fMRI could easily be added to HED, but many of the tags related to relevant experimental paradigms and events are already present in HED 2.0.

Neural ElectroMagnetic Ontologies (NEMO) is an ontology and collection of associated tools for annotating trial-averaged ERP time series extracted from EEG data (Frishkoff et al., 2007, 2011; LePendu and Dou, 2011). ERPs are obtained by averaging a group of data epochs of a fixed length all time-locked to the presentation of a given stimulus type. The NEMO project focuses on modeling ERP patterns at particular scalp regions, but also allows annotation of stimuli and other events as well as the experimental design using a controlled but limited vocabulary. The NEMO event ontology, based on the Neuroscience Information Framework (NIF) Standard Ontology (Bug et al., 2008) is based on the concepts of continuant and occurrent entities. Occurrent entities unfold in time, while continuant entities persist and maintain their identity through time (Arp et al., 2015).

The Cognitive Paradigm Ontology (CogPO) (Turner and Laird, 2011) was initially created to annotate experiments and associated publications for BrainMap (Laird et al., 2005; brainmap.org), a database of results from functional and structural neuroimaging experiments. CogPO consists of a vocabulary for describing stereotypical stimuli, instructions, and subject responses in human behavioral experiments. NeuroLex (Larson and Martone, 2013) is an online community effort to organize neuroscience nomenclature ranging in scale from neuron features to brain regions, including behavioral paradigms, using the Semantic MediaWiki (Krötzsch et al., 2006). The Semantic MediaWiki framework supports export to RDF (Resource Description Framework), making the results query-able for semantic relationships.

Another important neuroscience knowledge-building effort is the Cognitive Atlas (Poldrack et al., 2011), which seeks to define concepts, tasks, and disorders as well as the relationships between them. Concepts refer to mental representations or processes such as *visual color discrimination* or *spatial selective attention*. The Cognitive Atlas identifies tasks using stereotyped experimental categories such as *attention switching task*. Tasks have conditions, contrasts (condition comparisons, etc.), and indicators (measures of performance or results). The tasks link to relevant mental process concepts that specify how particular experimental contrasts index the associated processes. Tasks also link to collections that contain data using these tasks. Again, the Cognitive Atlas annotates at the experiment level and does not explicitly deal with experimental events.

The value of efforts to standardize data descriptions and event annotations to allow sharing and meaningful re-analysis and meta-analysis of neuroscience data has been demonstrated in a number of applications (Bigdely-Shamlo et al., 2013b; Ferguson et al., 2014). However, our experience has shown that while the existing ontology-based frameworks permit some documentation of experiment data at the cross-participant (study) level, they do not allow straightforward annotation of events in modern, complex laboratory, or real-world scenarios because of their complexity and the need for fine-grained detail in annotation. After identifying a collection of datasets that have been HED tagged and are in EEGLAB format, researchers can use the *HEDTools* to epoch the data across collection, irrespective of the studies contained in the collection. Researchers can use the epoched data for traditional ERP, ERSP, or time-locked classification and regression.

Many types of data analysis including transfer learning and other forms of meta-analysis can directly benefit from event annotation, the common starting point being finding similar events across some set of studies. This activity can be easily achieved using HED-annotated data with minimal programming effort in a wide range of programming languages, since it only involves simple string manipulation and matching. In particular, an “EEG Search Engine” powered by HED has been proposed (Bigdely-Shamlo et al., 2013a). Since HED describes multiple aspects of each event, it facilitates more thorough analysis of experimental factors relating experience to behavior and supporting brain dynamics via general linear modeling for statistical parametric mapping (SPM) (Friston et al., 1990, 1994), and regression (LIMO) (Pernet et al., 2011).

The HED 2.0 specification contains enough details to generate a formal ontology, e.g., in OWL format. Connecting HED with Linked Data is a worthwhile effort, which has already begun. However, most neuroscience researchers are more interested in finding similar events and studies than having a Linked Data representation, especially since Linked Data concepts are generally complex, tools to work with these technologies have a steep learning curve, and there are not yet extensive EEG open archives available on line. This is why we believe that the simplified interface provided by the HED syntax and its minimally-restrictive semi-sturctured tagging scheme is well positioned to foster the rapid adoption of neuroinformatics technologies in the fields of EEG analysis and BCI design. HED tagging is currently supported by the ESS, which focuses on the creation of containerized EEG collections that are self-contained and have standardized metadata (Bigdely-Shamlo et al., 2016). However, HED is not specific to ESS and can easily be incorporated as metadata to document events for data formatted following other standards.

We have built the HED 2.0 vocabulary through several iterations to make sure it is consistent and contains enough nodes to provide detailed information for events we encountered in tagging 9 real-world and 13 controlled-setting EEG studies. We observed that given a few hours of training most researchers are able to correctly and consistently tag many types of experiment events. There are, however, unique cases that require more deliberation and expertise. User comments from researchers outside our laboratories indicate that HED tagging is relatively straightforward, especially once an example of a particular paradigm has been developed.

HED tags are associated with events regardless of how the events were generated or the type of signal being tagged (EEG/MEG/fMRI time series, source, or time-frequency feature). In addition, HED tags can provide searchable overview annotations for an experiment as a whole and the ability to readily assess the frequencies of different types of events in an experiment. Researchers can also create events based on results of analyses (such as classifier outputs or to mark intervals within which some threshold is exceeded). We anticipate adding some basic tags to capture this type of annotation. Users can also add custom tags of specific interest.

In future work, we will be focusing on three areas for software development: handling of deeper parenthesis levels, better parsing of HED syntax for matching, and more integrated tools for performing analysis. The decision to limit HED syntax to one level of parentheses forces expression in terms of simple sentences, which we view as an advantage. However, there are situations where compound clauses, as expressed by more deeply nested tag sentences, would be advantageous. Our tools for matching and time-locked epoching based on tags work well with a single-level of parentheses, but will need enhancement for uses that are more complex. Another area of future work is the development of post-processing infrastructure to assign a state to each time point in an experiment based on event onsets, offsets, and durations. This infrastructure will enable a much more sophisticated assessment of user state and the interaction of events.

We are currently performing several large-scale, across-studies statistical, and machine learning analyses using HED tagged data. As more members of the dynamic brain imaging community adopt HED tag annotation of events, researchers will be able to determine whether similarly tagged data from other experiments share common brain dynamics. This question is fundamental to whether a given result is generalizable, an essential component of research reproducibility. We are currently working on a number of “case studies” that should make entry into the HED user community more attractive and straightforward. The increased pressure to share data by funding agencies, as well as the credit and extended data legacy provided by standardized annotation, are additional incentives for researchers to join in this effort.

The HED tools rely only on the specification of a vocabulary specified by an XML file. The HED 2.0 vocabulary file is available at https://github.com/BigEEGConsortium/HED/wiki/HED-Schema. All the tools developed in support of HED are also freely available at hedtags.org. Extensive user manuals for all of the tools are available via links from this site. *HED Tagging: A strategy guide for HED Annotation*, included as Supplementary Material to this paper, is also available at hedtags.org where updated versions of this guide will appear. An updated EEGLAB plug-in, CTAGGER, for tagging has been released and will soon be introduced to the larger EEGLAB community.

## Author contributions

NB helped conceive the idea, worked on the software, analyzed data, and participated in the writing of the manuscript. JC worked on the Matlab and Java software and helped analyze the data. SM helped conceive the idea and participated in the writing of the manuscript. TR was the initial developer of the CTAGGER user interface. CL wrote the Python version of the validation tools and worked on data integrity. MM contributed to the development of the HED hierarchy and its mapping to EEG. KR helped conceive the idea and participated in the writing of the manuscript, worked on the software, analyzed data, and participated in the writing of the manuscript. All authors read and agreed to the manuscript.

### Conflict of interest statement

The authors declare that the research was conducted in the absence of any commercial or financial relationships that could be construed as a potential conflict of interest.
