# Supplementary material for: Hierarchical Event Descriptors (HED): Semi-Structured Tagging for Real-World Events in Large-Scale EEG
Source: Front Neuroinform. 2016 Oct 17;10:42. doi: 10.3389/fninf.2016.00042 (PMC5065975; doi:10.3389/fninf.2016.00042)
Supplement: Supplementary file 1 [file Presentation1.PDF]

HED tagging (HED version 2.0)

A strategy guide for HED annotation

Analysis of event-related brain dynamics in human brain imaging experiments attempts to measure and model our brain dynamics in response to challenges posed by environmental events whose times of occurrence can be observed and recorded. These include presentations of sensory stimuli and, also, moments when expected stimuli are not presented, participant motor actions, as well as timed decisions *not* to act, and any other environmental or psychophysiological events (heartbeats, eye blinks, alpha bursts, etc.) whose times of occurrence can be observed and recorded – whether this be done during or after the experiment.

This document gives a simple overview of how to add exact descriptions to (i.e., how to annotate or tag) the experimental events whose times of occurrence are marked in your brain imaging datasets. To do this in a consistent yet user-extensible manner, we have developed the Hierarchical Event Descriptor (HED) schema and associated software tools. The HED schema is available online at:

http://www.hedtags.org/schema

The *http:www.hedtags.org* website also has links to relevant papers, user manuals and other documentation for the associated tools. Here we explain how to tag events using HED version 2.0. The HED framework has been developed for application to EEG brain imaging, but may also be applied to other brain imaging (MEG, fNIRS), multimodal (a.k.a, mobile brain/body imaging), physiological (ECG, EMG, GSR), or purely behavioral data. HED tools for MATLAB (The Mathworks, Inc.) and open source EEGLAB (*sccn.ucsd.edu/eeglab*) environments as well as xdf (*github.com/sccn/xdf*) data formats already exist. We expect others may soon follow.

We refer to the process of providing exact descriptions of events recorded in your data as 'HED-tagging.' The goal of HED-tagging is to describe precisely the nature of the events of interest occurring in the experiment using a common language, so that:

a. You and/or other researchers can better understand the experience of the participant,

and

b. Data analysis and meta-analysis processes can more easily and flexibly compare events (and brain responses to events) in these and other datasets.

This process will make it much easier for you to share your data and to insure that the value of the data you have worked so hard to collect endures, allowing your group and/or others in future to continue to extract new information from it – while also facilitating whatever analysis you may first apply to it.

Most brain imaging datasets include a list of recorded events that holds two essential types of information, the event latency and the event type. Typically, event types are recorded using cryptic alphanumeric codes.

> For example, in EEGLAB (*sccn.usd.edu/eeglab*), a signal processing environment running on MATLAB (The Mathworks, Inc.), the *EEG.event* structure array for an EEG dataset always includes two fields, *EEG.event[n].latency* and *EEG.event[n].type*.

Assume you have a dataset containing events of three types, originally designated by brief event codes *'CodeA'*, *'CodeB'*, and *'CodeC,'* and that you know (or can still determine!) what type of event each refers to. You can then describe these events more completely using HED tags. You may select the appropriate tags by using the CTAGGER GUI or you may type the event codes and known tags for each event type into a spreadsheet. In either case, HED tools can then map the tags to event codes in all of the EEG datasets in your experiment. In either case, *CTAGGER* and other HED tools can then map the tags to event codes in all of the EEG datasets in your experiment or produce tagged event lists in various formats. (See the *CTAGGER* user manual for details.)

The primary use of CTAGGER is to provide a simple user interface for annotating event types and to assist users in navigating the HED hierarchy and finding the right match for describing events. Novice HED taggers should use the CTAGGER GUI until they become familiar with the HED tag hierarchy and how to describe events in HED. Experienced taggers might find it still quicker to fill in an event type in a HED tag spreadsheet whose validity they then check using CTAGGER and other HED validation tools. After checking to make sure that all of the tags are valid, a researcher can then use *CTAGGER* to store the HED tags permanently in the dataset or in the entire collection if the same event codes appear.

> In EEGLAB, the HED tags are stored in the *EEG.event* structure array in two different fields. HED tags associated with a particular event type appear in the *EEG.event.usertags* field, while tags specific to that event appear in *EEG.event.hedtags*. Processing tools merge these lists for processing.

You may also instruct the experimental control script that collects and stores your data to insert appropriate HED tags whenever the control program produces or detects a stimulus, participant response, or other event. In future, we hope that common environments for creating experiment control scripts will support and encourage specification and automated saving of HED tag event descriptions with the recorded data at time of data collection. In this case, CTAGGER can later be used to tag events marked in the data by researchers *after* data collection – for example, events found in the session video ('yawn') or in the other experimental data ('sleep spindle').

Following, we break down the tagging process into separate steps and discuss which tags to use for what kinds of events. The most important overall goal in

HED tagging is to provide all the information that future analysts (e.g., you in future, as well as your associates, your collaborators, and your data heirs) will need to perform further meaningful analyses. Note that limiting the depth and completeness of the event tags to just that information you plan to use in a first-pass analysis can be short-sighted. When tagging events in your data, try to think more generally about the many analyses your data could support or contribute to – not just those questions you *first* want to answer about it.

Currently, most previously examined EEG data has nearly no practical use or value; HED tagging represents an important step towards more correctly valuing the wealth of known and yet unknown information contained in your carefully collected new and old data.

Basic HED tag syntax

Before getting into more details, we give an example of a "finished" annotation. This example shows a complete HED tag string for an event code representing a stimulus consisting of a fixation cross appearing at screen center. Certain elements appear in bold for readability in the example and are not part of HED.

Example:

> *Event/**Category**/Experimental stimulus,*
> *Event/**Label**/CrossFix,*
> *Event/**Description**/A cross appears at screen center to serve as a fixation point,*
>
> ***Sensory presentation**/Visual,*
> ***Item**/Object/2D Shape/Cross,*
> ***Attribute**/Visual/Fixation point,*
> ***Attribute**/Visual/Rendering type/Screen,*
> ***Attribute**/Location/Screen/Center*

[KAY: Here insert a figure showing selection of a portion(?) of this tag string in an EEGLAB CTAGGER application? -sm]

A *HED tag string* specifying an event annotation (as above) consists of several *tag paths* (or, simply, tags) separated by commas. The key idea is that these tag paths are not arbitrary. Rather, tag paths are hierarchical pathways through a predefined tree of terms, the HED 2.0 Schema (*http://www.hedtags.org/schema*) that provides the root terms (above: *Event, Category, Label, Description, Sensory Presentation, Item, Attribute,* etc.) of the HED 2.0 annotation language. Each tag or tag path is essentially a path through the HED tag hierarchy starting from a top-level node of the HED schema, for example: */Item/Object/2D Shape*. Since this hierarchy is extensible at its outer edges (leaves), you can enhance your annotations with as much detail as you like.

Since HED annotations consist of tag paths separated by commas, do *not* use commas or parentheses inside a tag or when specifying a numeric value (for example, '1,946'), though you may use semicolons or periods. (Note: Including a comma in an event description tag is a common error). CTAGGER and other HED validation tools will check your tags for syntactic correctness and will assist you in making any needed changes. You may omit the leading '`/`' if you wish, and need not adhere to letter case: in the example above, the tag string *`item/object/2d shape/CROSS`* would also be allowable.

The annotation process

The process of HED tagging your events (as in the above example) has five basic steps. After identifying the events associated with a dataset, for each type of event in your dataset you must then:

STEP 1: Specify the **category** of the event.

This is required and should always be your first step because it sets the stage for the types of tags you will need to complete the annotation.

`/Event/`**`Category`**`/XXX`

Events recorded in traditional laboratory EEG experiments generally fall into two (`XXX`) categories. **You need to select an `XXX` from these available options:**

1. **Stimulus presentation events.** These events mark moments when some sensible change occurs in the participant's environment. Typically, stimulus events mark (sudden) presentations of visual, auditory, or tactile ('present brief shock') stimuli or indicate stimulus stream onsets ('music begins to play'). The HED category path for such events is:

   *`/Event/Category/`**`Experimental stimulus`***

2. **Participant response events.** These events mark moments at which the participant responds to and/or acknowledges a stimulus event, in the past typically by pressing a finger response button. Technically, a response event signals one stage of a participant movement (often the moment an already accomplished finger button press is registered by the button system), but response events may also mark any defined stage of a participant action (such as the start of a steering correction in response to a vehicle perturbation in a driving simulation). The HED category path for such events is:

   *`/Event/Category/`**`Participant response`***

Other event categories. Other events mark moments in which something occurs that is neither a stimulus presentation nor a participant response. You may also use HED tags to document events that occur when:

a. The experimental control program performs some action not immediately perceivable by the participant, such as beginning a new task or stimulus block:

*/Event/Category/**Experiment control**/*

b. Something happens or changes in the environment:

*/Event/Category/**Environmental**/*

c. Something goes awry:

*/Event/Category/**Technical error/***

d. The participant misbehaves (for example by not following the instructions to drive in the correct lane in a driving experiment or by not giving one of the specified responses):

*/Event/Category/**Participant failure/***

e. Something completely unrelated to the experiment design occurs that you think might influence the data (for example, when in a 'real-world' experiment a loud airplane flies overhead or a passerby asks the participant whether they would like a cup of coffee):

*/Event/Category/**Incidental***

(Note: You might instead choose to annotate these events as */Event/Category/Technical error* if you think they may disrupt the data).

Finally, when you know of no appropriate category for an event you want to document:

*/Event/Category/**Miscellaneous***

STEP 2: Enter a short **label** (shorthand name) for this event type.

*/Event/**Label**/XXX*

The label itself (above, *XXX*) cannot be longer than 20 characters

*/Event/Label/**Red square***

You may use the already-existing data event code for this, but sometimes (especially for numeric codes required by some experiment control systems) it is better to use a more interpretable label, such as *3_ButtonPress* rather than just the meaningless event code *3*:

*/Event/Label/**3_ButtonPress***

Again**,** each event tag string must have one (and only one) */Event/**Label*** tag**.**

STEP 3: Enter a readable text **description** of the event.

An event description tag path is required for every event.

*/Event/**Description**/XXX*

Here, *XXX* can be a very detailed text description of this event type, **but must not contain any commas**. Description tags are not designed to be interpreted by computer programs, but instead to help users of the data to readily understand the nature of each data event and to see the logic involved in selecting its HED tags. You can import descriptions from your experimental notes or proposal. */Event/Description* tags help guide the rest of the HED-tagging process. Entering a clear, coherent description of the event type may help ensure that you annotate any potentially important attributes of the event by appropriate HED tags.

STEP 4: Add one or more descriptive tags appropriate for the event category and the nature of the particular event. Record relevant attributes of the event in the form of one or more event */**Attribute**/* tags:

Tags for **Stimulus presentation** events should capture the following information:

a. **How** the sensory stimulus was presented
b. **What** sensory stimulus was presented
c. The expected **import** of the stimulus presentation to the participant

For example, consider a stimulus presentation event in which a red square is presented (suddenly) in the center of the participant's computer monitor, and is expected to be 1) seen by the participant and 2) recognized by the participant as a relatively infrequent 'target' event prompting a designated behavioral (button press) or mental (event counting) participant response. The traditional approach is to designate a stimulus type (implicitly, a stimulus presentation event type) (e.g., type "square" or simply type "2") and to design appropriate analysis scripts and procedures that take into account the expected import of and participant responses to such "square" type events.

Such a limited approach is insufficient when you want to share your previously collected data with new students or collaborators or when you want to perform data meta-analysis, comparing EEG measures across more than one study. For example, one might ask, "Do brain responses to square object presentations differ in any way from responses to round object presentations?" Rather than design and carry out a pilot experiment to answer this question, given a base of HED-tagged datasets, some involving presentations of "square" stimuli and others, of "round" stimuli, you might glean a preliminary answer from (proper) statistical

meta-analysis of the combined data. If the results were positive and the question of strong enough interest, you might then propose an experiment to confirm or deny the hypothesis generated by the exploratory statistical study.

a. For stimulus presentation events, the minimal **"how"** tag is:

*/Sensory/Presentation/**Visual***

However, you could also document the fact that the stimulus was presented on a 2D monitor rather than in the 3D "real world" or in some virtual world:

*/Sensory/Presentation/Visual/**Rendering type**/Screen/2D*

b. A minimal **"what"** tag here is:

*/Item/**2D shape**/Rectangle/Square,*

Use tags under the */Item* node to specify objects (*Car*, *Animal*, *Food*) and 2-D or 3-D shapes and patterns.

You may also document stimulus:
color: */Attribute/Visual/**Color**/Red*,
location: */Attribute/**Location**/Screen/Center*,
size: */Attribute/**Size**/Length/2 cm,*
relative probability within its event stream:
*/Attribute/**Probability**/0.15*

Notice the use of units in the size attribute. HED specifies default and allowable units for attributes with physical dimensions such as size and the HED tools automatically check these. (See Section 2.6 for more details.)

c. If here we expect the participant to "see" the stimulus when it is presented, the minimal participant **"Import"** tag should be:

*/Participant/**Effect**/Visual*

Assumptions and caveats

The standard setup in traditional experiments assumes that subjects experience every stimulus, so a plausible assumption might be that a "**how**" specification:

/Sensory/Presentation/Visual

implies that the participant experienced the effect:

*/Participant/**Effect**/Visual*

However, in more complex, high event-rate scenarios including real-world neuroimaging studies, even truly awake participants may experience many distractions and may in fact *not* perceive all the presentation events.

More importantly, simply tagging a presentation as '*Visual*' does not capture the key elements of the experiment. In our example, the low-probability red square (occasionally presented within a stream of other centrally presented stimuli) should be expected to elicit a notable EEG 'oddball' response. If so, we should also specify:

*/Participant/Effect/**Cognitive**/Oddball*

when we have a reason to assume this (e.g. when subject also presses a button indicating their having recognized the target stimulus – or even when it seems too hard for the participant to miss). Further, if the participant is asked to look for and respond to red squares, we should specify:

*/Participant/Effect/**Cognitive**/Target*
*/Participant/Effect/**Cognitive**/**Cue***

2. Participant response events: Participant response events are usually simpler to tag than stimulus events and often mark a participant motor action. For example, if the subject presses a finger button, either in response to an earlier stimulus or mentally finishes a puzzle solution:

*/**Action**/Button press*

If we want to document this response in more detail:

*/**Action**/Button press/Keyboard/Key/h*

This event might be given the label:

*/Event/Label/**Pressed h***

although a label noting the meaning of the action, such as ***/Response: High*** might be more useful. If the participant always presses a button with their left index finger, we can also document this detail:

/Participant/Effect/Body part/Arm/Hand/Finger/Index,
/Attribute/Object side/Left

3. Other categories of events: Tag other types of events in as much detail as you think is necessary to convey information potentially needed for both near and far term analyses and meta-analyses of the data.

STEP 5: Validate the tag(s), adjust, and retest as necessary

Once you have tagged an event of each type in the data, you should run the CTAGGER or other HED Tools validation functions to make sure that the tags have correct HED syntax. Make any needed changes and revalidate until your HED annotations all pass the syntax validity checks.

2 Some guidelines

2.1 Onsets and offsets

Stimulus offset events. Suppose an event is a part of an onset-offset pair: for example, an event for image onset (when the object appears) and another event for image offset (when the image disappears). **Use the *same* event label and event description for both the onset and offset events**. Instead, use `/Attribute/`***`Onset`*** and `/Attribute/`***`Offset`*** tags to specify the action. Using the same event label and event description for both stimulus onset and offset allows easier automated discovery and processing of onset-offset event pairs. If you use the `/Attribute/Onset` tag, you should add a matching offset event with an offset attribute tag. Do not include the words "onset", "offset", "start", "end" or similar words in the event label. However, some onset events have qualitatively different from the corresponding offset events. For example, the beginning of a walking stride is typically coded as a *`/Toe Off`* event and the end of the step a *`/Heel Strike`* event – these should be tagged as events of different types.

2.2 Event durations

Some events have a fixed (and known) duration, for example, an image may appear on display for 500 ms before disappearing. Here, again, you may create two events, separated in time by 500 ms, to indicate the beginning and end of such events, using *`/Attribute/`****`Onset`*** and *`/Attribute/`****`Offset`*** tags, respectively. Alternatively you may use the tag:

*`Attribute/`****`Duration`****`/500 ms`*

2.3 Sensory presentation versus participant effect

HED 2.0 allows you to define events that no participant could possibly perceive as a stimulus (such as when a truck in a screen animation temporarily moves behind a building). Events like this are useful for making sense of complex experiments, for inferring what the participant(s) are attending to or imagining. In a sense, HED 2.0 is objective, describing what happens during the experiment independently of what affects or is perceived by the participant.

Use tags under ***`/Sensory presentation`*** to specify the sensory presentations of the item specified. For example, in an experiment in which a participant views a computer animation, an approaching car in a scene shown on the monitor may involve auditory presentation (its approaching rumble before and as it comes into view) as well as visual presentation (its coming into view). In the latter case its HED string should be:

`/Item/Object/Vehicle/Car,`
*`/`****`Sensory presentation`****`/Auditory,`*

*/Sensory presentation/Visual*Attributes are somehow special, since you can combine them to provide more information about nodes at different parts of the hierarchy. For example:

/Attribute/Language/Unit/Sentence/Full

can describe a sentence presented visually or aurally.

If you have enough evidence to assume that the participant perceives a stimulus (or at least have no evidence to the contrary), you should add one or more */Participant/Effect* tags specifying the type(s) of experiential effects (cognitive, visual, auditory, etc.).

2.4 HED parentheses

HED 2 allows only one level of parentheses --- you cannot use nested parentheses. Use parentheses to group attributes with the items they modify. For example, if a stimulus consists of simultaneous presentation of a red square and a blue circle, you would tag:

```
(/Item/Object/2D shape/Rectangle/Square,
 /Attribute/Visual/Color/Red),

(/Item/Object/2D shape/Ellipse/Circle,
 /Attribute/Visual/Color/Blue)
```

2.5 IDs

Complex experiments can have multiple objects interacting with multiple participants. HED supports IDs for both items and participants. You can assign detailed properties to an item along with an ID. We could describe the red car when it first appears and use */Item/ID/RedSquare* to identify the car in subsequent events.

```
(/Item/ID/RedSquare,
 /Item/Object/2D shape/Rectangle/Square,
 /Attribute/Visual/Color/Red,
 /Attribute/Location/Screen/Center)
```

HED IDs identify objects or participants with global scope. The tag path:

```
/Item/ID/RedSquare
```

in two events identifies the same object. HED also supports local IDs that are only valid within a particular event:

```
/Item/ID/Local/Square1
```

2.6 HED clauses

Parentheses also define HED clauses to describe more complicated event structure. For example, if the system control software had previously displayed a car animation in a 2D window, but participant just now saw the car, as inferred by an initial saccade, we may want to record that, at the succeeding fixation, "Participant ID1 saw the Red Car." A **HED clause** always has a single level of parentheses and is in one of two forms: simple or transitive. A simple clause is in the form:

```
(subject ~ verb)
```

(Note: a more formal term for verb is predicate). When the verb takes an object (as in: "Participant ID1 saw the Red Car."), use the transitive form:

```
(subject ~ verb ~ object)
```

A HED clause expressing that participant with ID 1 saw the displayed car is:

```
(/Participant/ID 1
 ~
 /Participant/Effect/Visual
 ~
 /Item/Object/Vehicle/Car,
 /Item/ID/RedCar,
 /Attribute/Visual/Color/Red)
```

Since in this example we are not assuming that the participant sees the car when it first appears, we should have other events marking the appearance (onset) and disappearance (offset) of the item */Item/ID/RedCar*. We could describe the red car when it first appears and use */Item/ID/RedCar* to identify the car in subsequent events.

As you can see, this HED "clause" is made of three parts, separated by the special character tilde ('~') and enclosed in parentheses. The object (the third part of the HED clause) is not present in all cases, as in the following which records a moment in which the car's path is perturbed.

```
(/Item/Object/Vehicle/Car
 ~
 /Attribute/Object control/Perturb)
```

2.7 Event repetitions

Sometimes, by design the same type of event is repeated in close succession – though participant brain dynamics during and following its repetitions may turn out to be quite different. Suppose, for example, a participant looking for a target object on a display screen makes multiple eye saccades to a target object within two seconds of target appearance. It is likely that the first saccade elicits a target recognition response while succeeding saccades may not. When event repetitions are a standard part of the experimental protocol, you may use:

/Attribute/Repetition/#number

tags to designate which repetition an event represents. The first repetition should have the */Attribute/Repetition/1* tag, the second one */Attribute/Repetition/2* and so forth.

2.8 HED unit classes

HED enforces units for many numeric attributes such as length, currency, angle, frequency, and many more. Each unit class has default units, which HED validation tools can supply. The HED schema also specifies the units that are allowed for a particular numeric attribute.

2.9 Adding nodes to the hierarchy

You can add new nodes below any lowest (leaf) node in the HED string hierarchy to record further details. For example, the tag path:

/Item/Object/Vehicle/Car/Toyota/Camry/2012

records finer details about a car image shown to the participant in an experiment. Allowing free expansion of the leaves of the HED hierarchy permits taggers to provide as much detail as possible without cluttering the root hierarchy. Here, for example, it would not likely be useful to add every possible car make and model to the HED tag hierarchy!

This ability to extend the HED term hierarchy in a flexible way makes HED a semi-structured language and is connected to the name for 'CTAGGER' in which the 'C' stands for Community. We hope that the ready extensibility of the HED hierarchy will lead to more rapid adoption and elaboration of HED tag terminology by a large community of users. CTAGGER supports use of the root HED tag hierarchy as well as, *by user choice*, its accepted community extensions, its local (lab) extensions, and/or its personal extensions. Here, the user's choice may depend on the purpose of the annotation.

You may also add new HED tags at a few higher-level (non-leaf) locations in the HED tag tree hierarchy. The online HED 2.0 schema indicates these locations by the attribute *{extensionAllowed}*.

2.10 Custom tags

Some experiments require an experiment-specific annotation hierarchy, for example a specific hierarchy of object tags that does not seem to fit exactly into HED, or whose inclusion in the community HED schema would be unlikely to be viewed as a generally useful addition. In such cases, in addition to using HED nodes to maximally describe different aspects of the event (including extending HED tag descriptions at the lowest "leaf" nodes), you can capture a HED-parallel hierarchy under the */Custom* string node. HED places no restrictions on HED strings defined under */Custom*. HED tools match tags under */Custom* using

the same strategies as for other tags in the hierarchy. For example, */Custom/Dance/Waltz* will be considered a subtype of */Custom/Dance*.

You should avoid using /Custom for annotating events that share some conceptual equivalence to events in other studies and that you want to be discoverable by others. The more */Custom* tags you use, the less your annotations will have in common with those of other studies. Therefore, we strongly suggest you not use */Custom* strings as the sole descriptors of your events. The order of */Custom* strings does not matter, but as a matter of style it would be better if, when needed, they are put at the end of your tags.

3 More examples

3.1 The vehicle in a driving simulation experiences a perturbation

The following is an example annotation describing an event for a perturbation in the position or path of an animated vehicle during a driving simulation that tests driver reaction time:

/Event/Category/Experimental stimulus,

/Event/Label/PerturbLeft,

/Event/Description/The vehicle experiences a sudden push left which continues until the user responds by correcting the vehicle position to the center of the lane,

/Item/Object/Vehicle/Car,
/Attribute/Object control/Perturb,
/Attribute/Direction/Left

You should also add a HED clause to tag the subject's perception of the perturbation, as illustrated in the previous section. In this case, the presentation is visual, since the subject can only know that the perturbation has happened by seeing an unexpected change in simulated vehicle lane position:

/Sensory presentation/Visual

Similarly, the participant effect is also visual:

/Participant/Effect/Visual/Perturbation

3.2 Example event with two objects and two participants

Suppose two objects are visible in an experiment: a simulated truck (item ID 5) and a simulated pedestrian (item ID 8) appearing on the passenger side of the vehicle. One participant (participant ID 1) only hears the truck, while the second

participant (participant ID 2) both sees and hears the truck. The pedestrian is visible on the screen, but we do not know if either participant saw it.

```
(/Item/ID/5,
 /Item/Object/Truck,
 /Sensory presentation/Visual,
 /Sensory presentation/Auditory),

(/Item/ID/8,
 /Item/Object/Complex/Person/Pedestrian,
 /Attribute/Object side/Passenger/,
 /Attribute/Object Side/Reference Object ID/5,
 /Sensory presentation/Visual),

(/Item/ID/5,
 /Participant/ID/1,
 /Participant/Effect/Auditory),

(/Item/ID/5,
 /Participant/ID/2,
 /Participant/Effect/Visual,
 /Participant/Effect/Auditory),
```

3.3 Saccades

You can encode saccade events as participant responses events or as incidental events, depending on the relationship to the task. If there is an onset event, there should also be an offset event. We only show an example of saccade tagging here.

```
/Action/Eye saccade/Onset,
/Attribute/Location/Screen/Top/7 px,
/Attribute/Location/Screen/Left/230 px,
/Attribute/Direction/Angle/-185 degrees,
/Attribute/Distance/256 px
```

3.4 Footsteps

In an experiment in which the subject walks outdoors, we may want to mark and tag events corresponding to important events in the participant's stride:

```
/Action/Walk/Stride/,
Attribute/Onset,
/Action/Body part/Leg/Feet/Toes,
/Attribute/Object side/Right,
/Attribute/Location/Real-world
coordinates/Room/xyz/12.3 8.5 6.7
```

3.5 Reaching to touch

Here is an example of tagging the details of an event involving reaching to touch:

```
/Action/Reach/To touch,
(/Attribute/Object side/Left,
/Participant/Effect/Body part/Arm),
/Attribute/Location/Screen/Top/70 px,
/Attribute/Location/Screen/Left/23 px
```

4 Final remarks

This document continues to evolve with additional examples as we encounter interesting tagging challenges. You can always find the latest version at *hedtags.org*.

Kay Robbins, Nima Bigdely-Shamlo, Scott Makeig, Makoto Miyakoshi, and Mike Dunkel have contributed to this document.
