# Supplementary material for: Hierarchical Event Descriptors (HED): Semi-Structured Tagging for Real-World Events in Large-Scale EEG
Source: Front Neuroinform. 2016 Oct 17;10:42. doi: 10.3389/fninf.2016.00042 (PMC5065975; doi:10.3389/fninf.2016.00042)
Supplement: Supplementary file 2 [file Presentation2.PDF]

Some user comments from researchers using HED tags

1) Do you feel that the HED tagging system captures the meaning of the events?

"Yes. The HED tagging system captures everything from the detailed description of objects and actions to the experimental context of each event. The system is very flexible and can describe the meaning of events in as much detail as desired."

"Yes. It seems, however, that there will often be details specific to an experiment that require unique tags to adequately describe the event. The HED tag "dictionary" will need to be ever expanding and if enough people use it, there will need to be a system for people to submit new items for inclusion."

"The use of existing tags for events in new data sets should be a thoughtful exercise, to ensure consistency in the application of tags to experimental activities. The HED hierarchy should be considered as a "living structure", in that it may require modification for new events that do not fit an existing tag definition."

2) Do you use the tools to support your tagging?

"Yes. We use the HED validator to ensure our tags are free of errors."

"I mostly build a set of tags in an Excel spreadsheet, keeping CTAGGER open with the latest HED tag hierarchy to use as a guide. I use this method because for the most part I'm building one set of tags from an existing set. If I was starting from scratch with a project unrelated to a previously tagged one I'd probably build the tags within CTAGGER. I always use the validation tools to validate a set of tags. We need a better system for a remote user to check on the current HED version and to download the xml file."

3) Does formulating the tags help you to clarify the meaning of the experiment?

"Yes. Formulating the tags helps me clarify the meaning of the experiment for future users of the data set and for myself. I came on board my project at a later stage and found some of the subtleties of the experiments were best understood by examining the existing HED tags and then formulating HED tags for additional events."

"Yes. The process of tagging events and explaining their definitions to others forces a clear understanding of the experimental data, and the events derived from them."

"Yes. I'm just processing other people's data and I wasn't involved in the data collection, so HED tagging helps me understand what was going on."

4) Is the tagging process getting any easier as you gained experience with it?

“Yes. Tagging has gotten easier with practice, but tagging is a straightforward process to begin with.”

“Yes. Each new data set that’s tagged adds to the contextual understanding of event definitions. Again, it helps ensure consistency when defining conceptual-level information.”

5) Other comments?

“In addition to the goals for using our HED tags with machine learning, HED tags are immediately useful for finding and categorizing our large number of events. I can't think of a better way one would navigate through the 41,000 events in our study.”

“Although and expanding body of researchers contributing to the hierarchy will enrich the tag set, there still needs to be limited control over formal definitions. A “tag committee” would be an effective mechanism for negotiating event definitions and the (appropriate) corresponding tags.”

“I think, if you want the HED tags to have a standardized meaning across multiple experiments and if many more people start applying the HED tagging process, that a thorough guide, essentially a HED tagging textbook, needs to be developed that details the grammar and syntax of HED tags. Explain and give clear guidance for things like using parentheses and tildes.”
